# An Integrated Perspective of Effort and Perception of Effort

**DOI:** 10.1007/s40279-024-02055-8

**Published:** 2024-06-23

**Authors:** Israel Halperin, Andrew D. Vigotsky

**Affiliations:** 1 Department of Health Promotion, School of Public Health, Faculty of Medical & Health Sciences, Tel-Aviv University, Tel-Aviv, Israel; 2 Sylvan Adams Sports Institute, Tel Aviv University, Tel-Aviv, Israel; 3 Departments of Biomedical Engineering and Statistics, Northwestern University, Evanston, IL USA; 4 Department of Neuroscience, Northwestern University, Chicago, IL USA

## Abstract

Effort and the perception of effort (PE) have been extensively studied across disciplines, resulting in multiple definitions. These inconsistencies block scientific progress by impeding effective communication between and within fields. Here, we present an integrated perspective of effort and PE that is applicable to both physical and cognitive activities. We define effort as the energy utilized to perform an action. This definition can be applied to biological entities performing various voluntary or involuntary activities, irrespective of whether the effort contributes to goal achievement. Then, we define PE as the instantaneous experience of utilizing energy to perform an action. This definition builds on that of effort without conflating it with other subjective experiences. We explore the nature of effort and PE as constructs and variables and highlight key considerations in their measurement. Our integrated perspective aims to facilitate a deeper understanding of these constructs, refine research methodologies, and promote interdisciplinary collaborations.

## Key Points

**Table:** 

The definitions of effort and perception of effort (PE) vary greatly within and between disciplines, hindering effective communication.
We propose an integrated perspective that defines effort and PE in a manner that is applicable to both physical and cognitive activities.
Our aim is to enhance understanding of these constructs, improve research methodologies, and foster interdisciplinary collaborations.

## Introduction

Effort and perception of effort (PE) are intuitive concepts. Individuals recognize that physical and cognitive activities, such as lifting objects and solving math problems, demand effort and are associated with PE. However, despite extensive work by philosophers, psychologists, neuroscientists, and exercise scientists, formal definitions of effort and PE vary widely across and even within disciplines [1–15]. A contributing factor to this inconsistency is the tendency to study effort and PE in isolation, segregated into physical or cognitive categories. Inconsistencies in defining concepts such as these can impede collaborative advancement and scientific progress [3, 11, 12, 16, 17]. Yet, only a few have attempted to integrate effort and PE across domains [7, 9]. Here, we propose integrating the definitions of effort and PE across physical and cognitive domains. Before covering the specifics, we outline what such a perspective offers.^1^

An integrated perspective of effort and PE provides two key advantages. First, jointly exploring these concepts can facilitate a deeper examination of their interconnectedness. For instance, studying the causes of physical and cognitive task failures becomes more insightful when effort and PE are examined together. Doing so can help us understand whether task failure is primarily attributable to maximal effort, maximal PE, or a combination of both. Second, distinguishing between effort and PE can reduce the ambiguity in the literature resulting from their conflation [12]. By defining effort as an objective process and PE as a subjective experience, we aim to mitigate this ambiguity.

Given their shared characteristics, defining effort and PE across physical and cognitive activities can be beneficial. We distinguish between physical and cognitive activities as follows: physical activities rely primarily on muscular contractions—e.g., walking, lifting, and throwing. Conversely, cognitive activities rely primarily on information processing, decision-making, and memory recall.^2^ This distinction is not binary but exists along a continuum, as most activities involve physical and cognitive elements. For instance, a soccer player sprinting while dribbling the ball (a physical act) simultaneously processes strategic game information and is required to make rapid decisions (cognitive acts). Even activities that seem to lean heavily towards one end of the continuum integrate both elements. For example, solving math problems often necessitates mediating one’s response through physical actions (e.g., pressing a calculator button), and lifting a heavy object involves cognitive actions (e.g., overriding the desire to stop). Thus, an integrated perspective of effort and PE can marry insights from seemingly disparate disciplines. We note that our primary background in exercise science naturally led us to focus more on physical activities.

This article is structured as follows: We begin by discussing effort in Sects. 2, 3, 4, 5. Within these sections, we explore different accounts of effort, propose and support a definition of effort, examine its nature as a variable, and conclude by outlining measurement considerations. We then move on to PE in Sects. 6, 7, 8, following a similar approach to that of effort. We briefly address the advantages of studying effort and PE together in Sect. 9 and evaluate the strengths and weaknesses of this integrated perspective in Sect. 10.

Before presenting our definitions of effort and PE, we want to clarify that the perspective we present is more of a philosophical position. The definitions we present are axioms—not hypotheses that can necessarily be empirically tested. These axioms may have both favorable and unfavorable properties and thus are subject to change over time with future research and discourse.

## Effort

### Accounts of Effort

Several comprehensive accounts of effort have been proposed: some conceptualize effort as applying forces against a resistive force to achieve a goal [6, 7, 19]; some frame effort as a mediator between individual abilities and task performance, given task demands [5, 20, 21]; and others characterize effort as utilizing resources to perform tasks [22–24]. Here, we expand upon the latter, resource-based account, as the basis for our integrated perspective. Our decision does not imply that this account is superior to others. Instead, since different accounts emphasize distinct aspects of effort, they do not necessarily conflict and may even be complementary. Since exploring different effort accounts is beyond the scope of this article, we refer the interested reader to Massin [6] for a detailed review of this topic.

### Resource-Based Accounts of Effort

Resource-based accounts conceptualize effort as utilizing resources to complete actions, with greater efforts requiring more resources. Before introducing a specific definition and its implications, we outline the reasons for selecting this account. First, we view resource utilization as a fundamental cornerstone of effortful actions in biological systems. Resource utilization underpins other notions of effort (e.g., force and mediation-based accounts), which require more resources to increase their endpoint (see the Appendix for further details). Second, this account is widely used in physical and cognitive effort research, signifying its utility and relevance [25–29]. Third, it can be naturally extended to PE, allowing us to maintain consistency within our perspective. Fourth, it is intuitive and easy to grasp and communicate.

Within the resource-based account, several definitions of effort exist, with two notable examples being the “mobilization of resources to carry out instrumental behavior” [24] and the “deliberate allocation of mental resources to overcome obstacles in goal pursuit when carrying out a task*”* [30]. In the following sections, we will introduce and justify a working definition of effort that builds upon yet diverges in several key aspects from these established definitions.

## Developing a Resource-Based Effort Definition

In this section, we propose a definition of effort as *the energy utilized to perform an action* and then unpack the meaning of its components. Definitions of key effort-related terms, which will now be covered, can be found in Table 1.

**Table 1:** Definitions of key effort terms

Term	Definition
Effort	The energy utilized to perform an action
Energy	Depletable substrates (e.g., adenosine triphosphate)
Action	The process of doing something, often but not necessarily to attain a goal
Effective effort	Effort that aids in achieving a goal
Ineffective effort	Effort that does not aid in achieving a goal
Instantaneous effort	The amount of energy utilized per unit of time
Cumulative effort	The sum of energy utilized throughout the activity
Maximal capacity	The maximum amount of energy that one can utilize in an action
Performance	How well one does an activity as assessed based on objective criteria
Absolute effort	The energy utilized in performing an action independent of one’s maximal capacity
Relative effort	The energy utilized normalized to one’s maximal capacity

### Energy as a Resource

In the broad context of resource-based frameworks, resources signify different things but typically fall into one of two broad categories: depletable or allocatable. For example, glucose is a depletable resource, whereas attention and memory (resources) can be allocated toward a task but are not necessarily depletable. Here, we adopt the former view of resources, identifying them as depletable substrates. We emphasize that in our definition of effort, energy encompasses both the total amount and the rate at which it is used, a topic we elaborate on in Sect. 5.2.

In the context of biological systems—our principal interest—energy primarily consists of adenosine triphosphate (ATP). This organic compound acts as the “molecular unit of currency” for intracellular energy transfer and biological processes [31]. ATP is depletable and renewable. The balance between ATP consumption and regeneration is primarily determined by the demands of the activity and the capacity to resynthesize ATP via various metabolic pathways [32]. Consequently, ATP levels can diminish when demand surpasses resynthesis capabilities. Energy utilization can be measured in several ways: directly, at the ATP level, or indirectly, using physiological outputs such as thermal energy production, oxygen consumption, and heart rate. Energy utilization can also be measured indirectly through performance. We refer to performance as how well an individual does an activity, as assessed based on objective criteria. Examples of performance include the time it takes to lift an object or react to visual cues (see Sect. 4.4 for a discussion on effort and performance).

#### Energy in Physical Effort

Decades of research have established a clear association between the intensity and duration of physical activities and energy utilization as measured via direct and indirect calorimetry, heart rate, and blood lactate [33–39]. This relationship is supported by well-established biological mechanisms [40, 41], where physical activities elevate metabolic demands, necessitating ATP consumption and regeneration.

#### Energy in Cognitive Effort

Ongoing debates center around the energetic “costs” of cognitive effort. Some argue that cognitive effort is mostly attributable to the allocation of computational processes rather than the depletion of energetic resources beyond baseline requirements [4, 42–44]. This viewpoint is supported by studies indicating stable peripheral blood glucose levels (a precursor of ATP) during cognitive tasks, implying the brain’s glucose consumption remains constant regardless of cognitive workload [4, 42–44]. However, a large body of evidence contradicts this conclusion [45]. First, in animal studies, cognitive tasks deplete brain glucose in specific brain regions [46–48], even without changes in peripheral blood glucose levels [49]. Second, human neuroimaging studies consistently report localized elevated blood oxygenation-dependent and positron emission tomography activity during cognitive tasks [50–54], which are proxies of metabolic activity [55, 56]. While the additional energetic costs of cognitive effort may be low compared to the baseline metabolic demands of the brain, it nevertheless seems to require some depletable resources [33].

#### Constraints of Effort

Some actions are limited by physical or computational constraints rather than by energy availability. For example, limited muscle mass may prevent someone from lifting a car rather than insufficient ATP. Similarly, cognitive computational resources may prevent someone from quickly counting backward by sevens rather than insufficient ATP. Although such bottlenecks do not fulfill our definition of effort, they may limit effort output. Consider a driving analogy: we view effort as the fuel utilized to maintain a certain speed. The diameter of the fuel line can limit fuel flow and thus speed, but the diameter of the fuel line itself is not an effort.

### Utilization

We prefer the term “utilized” instead of the terms “mobilized” or “allocated” used in other resource-based account definitions [24, 30]. At least theoretically, one can mobilize or allocate resources without using them, whereas utilization occurs while performing an action. We thus expect that the term utilized will aid in clarifying this issue in our proposed definition.

### Action

We define action as the process of doing something. Actions are often but not necessarily performed to attain a goal. Our preference for “action” over “goal” is as follows. First, goals are often defined with respect to a desired endpoint. However, effort can also be exerted during neutral and undesirable states. For example, people’s heart rate increases in anticipation of goal-oriented activity [57], such as an exercise session [58] or presenting in front of an audience [59]. While the elevated heart rate is not necessarily desirable and may even be counterproductive, we nevertheless view it as effortful. Hence, effort can be present without a clear goal, making “action” more inclusive.

Second, the beginnings and endings of goal pursuits can be ambiguous. This ambiguity can extend to the measurement of effort, as it relies on a clear understanding of when a goal-directed action begins and ends. However, pinpointing these moments can be challenging and overly restrictive. For example, consider the goal of going for a walk. There are various points at which the goal-directed action could be considered to begin, leading to different interpretations of when effort measurement should start: Should it commence when the commitment to pursue it is made, when walking toward the park where the walk officially begins, or the first step once reaching the walking course? To address this issue, using the term “action” allows for more flexibility in defining the start and end points, irrespective of a specific goal.

We want to emphasize that our use of “action” encompasses both goal-directed and goal-undirected actions. Unlike a definition focusing solely on the former, our approach considers all actions, regardless of whether a specific goal exists. In situations where a clear goal is evident, distinguishing between two types of efforts can be useful: effective effort, which contributes to achieving a goal, and ineffective effort, which does not. For example, in cycling, force effectiveness evaluates how much of the force applied to the pedals translates into propelling the bike forward. Assuming a cyclist’s aim is to maximize speed, the forces generating propulsion are effective efforts, while other forces, like radial forces, are ineffective efforts. For a similar example in the cognitive domain, see [60]. Though differentiating between these efforts can be empirically and conceptually challenging, when feasible, separating them has theoretical and practical value. Theoretically, it can deepen our understanding of effort expenditure processes. Practically, identifying the most performance-enhancing efforts can optimize training programs across domains.

### Does Effort Need to be Volitional?

When formulating the effort definition, we considered whether to include the term volition and decided against it. Volition is broadly defined as the ability to make choices and control one’s thoughts and actions [61–63]. Including volition in the definition of effort presupposes a clear distinction between organisms that can and cannot express volition—a matter of ongoing debate [62, 64]. By excluding it, we bypass these dilemmas and broaden the definition’s applicability, allowing for greater consistency in the term’s meaning across disciplines, from ethology to psychology. Another advantage of excluding the term volition is that it resolves the inconsistency in the meaning of the same actions performed with or without volitional control, such as blinking [65] and breathing [66]. Similarly, bodily movement can be generated voluntarily or involuntarily through reflexes and electrical stimulation [67]. Involuntary actions would not be considered effortful if volition is part of the proposed definition, while they would be considered effortful if the term was removed. Thus, to avoid the problems mentioned above and for the sake of consistency, we decided against including volition in our definition of effort.

### How Does Effort Differ from Energy Expenditure?

Since our definition of effort excludes the terms volition and goals, it is natural to question how it differs from “energy expenditure.” We acknowledge a considerable overlap between effort and energy expenditure, as both involve quantifying energy utilization. Rather than viewing this overlap as problematic, we see it as an opportunity for interdisciplinary knowledge exchange. Importantly, however, the terms have nuanced differences. Whereas energy expenditure is typically defined as the total amount of energy expended irrespective of specific actions, we define effort as the energy utilized to perform an action. Without this specification, it would simply be energy expenditure. Given that living beings continuously expend energy, it is reasonable to assert that effort is continuously exerted. However, the effort must be contextualized by a specified action; otherwise, our definition cannot be applied. Moreover, the two differ in measurement strategies: effort is commonly estimated in ways not used to estimate energy expenditure, with the relative effort being a notable example (Sect. 5.1).

## Effort as a Variable

### Effort as a Latent Variable

Effort is a complex process that depends on interactions between the performer’s various systems and the action at hand. Since this process is not entirely observable, we view effort as a latent variable. Effort can be measured to varying degrees, depending on the actions performed and the measurement tools. However, confirming what portion of the collected data reflects the effort of interest is challenging—i.e., relative to some baseline or an alternative condition. Therefore, conceptualizing effort as a latent variable that can be captured to a lesser or greater extent via proxies seems sensible.

### Operationalization of Effort’s Definition

If effort is conceptualized as a latent variable, it requires operationalization—i.e., conversion into a measurable variable. To do so, researchers and practitioners first need to decide on the action of interest they will use for their project. This means replacing the term *action* in the definition with a specific action, such as a lifting, running, memorizing, and reading activity (see Sect. 4.4 for a cautionary note). Then, they need to decide how they will quantify energy utilization for that action. This can be done using various measures, including oxygen consumption, heart rate, and electromyograms. Examples of operationalization of effort’s definition include the calories expended to perform a 5-km run, the forces applied to the ground to perform a countermovement jump, and the oxygen consumed to perform a Stroop test. While the latter examples use terminology that strays from the proposed effort definition, they align with its fundamental assumptions. The first part of each example indicates how energy utilization will be measured (e.g., physiological outputs or performance) and if the measurement is direct or indirect.^3^ The second part of each example specifies the action. We thus consider these examples consistent with the proposed effort definition despite the differences in terms.

### Effort as a Dependent or Independent Variable

Effort commonly serves as the dependent variable in research, for example, evaluating the impact of interventions on participants’ indicators of effort. While less common, effort can also be conceptualized as an independent variable. For instance, consider a stepwise incremental exercise test performed on a treadmill to assess maximal aerobic capacity [68]. During the test, the treadmill’s speed gradually increases until participants reach exhaustion [68]. Researchers often regard such manipulation as pertaining to the action’s difficulty (e.g., the speed). Yet, in this case, increasing difficulty necessitates greater energy expenditure and, therefore, effort. Thus, the manipulation of difficulty may be conceptualized as a manipulation of effort, positioning effort as the independent variable. While we are not suggesting changing this practice, there is merit in conceptualizing difficulty as effort, encouraging a deeper consideration of what the manipulated factor is and how it relates to effort.

### Effort and Performance

It is important to conclude this section with a limitation of resource-based accounts—they often use performance to infer effort. However, the relationship between effort and performance is multifaceted. For example, investing more effort to perform a task incorrectly, such as contracting the wrong muscle or contracting the correct muscle at an inappropriate time, will not necessarily enhance performance. There is also a ceiling effect beyond which additional effort will not improve performance. For example, exerting more effort will not enable someone to lift a truck if they lack the potential physical capacity to do so. Training interventions can improve cognitive [69, 70] and movement [71, 72] efficiency, whereby completing the same action requires less effort. Finally, performance can be influenced by PE, as discussed in Sect. 8. Nonetheless, empirical investigations consistently demonstrate associations between effort and performance [27–29, 73–75]. Thus, performance can often serve as a reasonably accurate indicator of effort, allowing us to exploit this relationship with minimal drawbacks.

## Measurement Considerations

Here, we discuss two important measurement aspects of effort: (1) absolute and relative effort and (2) time. Our focus on these specific aspects is driven by their relevance to our perspective. This discussion is not intended to be an exhaustive review of all variables related to the measurement of effort.

### Absolute and Relative Effort

One can express effort in absolute and relative terms. Absolute effort is the energy utilized in performing an action independent of one’s maximal capacity, which is the maximum amount of energy that one can utilize in an action. Absolute effort is ideally expressed in units of energy (joules or calories) or power (watts), but in practice, it is more commonly expressed in units like kilograms, meters per second, and beats per minute. To illustrate, assume that we prescribe two individuals to lift barbells loaded with 20 and 50 kg from the ground. Lifting the heavier load will require greater absolute effort. Since 20 and 50 kg are absolute quantities that do not account for an individual’s maximal capacity—i.e., the heaviest load they can lift once (1 repetition maximum [1RM])—they can be operationalized as “absolute effort.”

Relative effort is the energy utilized relative to one’s maximal capacity and is thus expressed as a percentage. However, since we do not know whether one truly utilized the maximum possible energy in performing an action (i.e., maximal capacity), in practice, to estimate relative effort, it is required to know the individuals’ maximal physiological output or performance in the specific action. This measurement should ideally be recent and conducted under conditions allowing for optimal performance. Returning to the earlier example, assume both individuals’ 1RMs were 100 kg and 200 kg, respectively. This means that lifting 20 and 50 kg will correspond to relative efforts of 20% and 50% of 1RM of one individual (i.e., 20 kg/100 kg) and 10% and 25% of the other (i.e., 20 kg/200 kg).

#### Absolute and Relative Effort in the Same Action

Differentiating between absolute and relative effort enables prescriptions of effort to accommodate inter-individual differences. For example, imagine a group of people required to lift a 20-kg bag while researchers measure their effort expenditure. Despite lifting the same load, participants exerted varied relative efforts. For some, the 20-kg bag might represent 40% of their 1RM, while for others, it might represent 80% of their 1RM. In such experiments, participants’ absolute efforts are difficult to interpret without knowing their abilities, limiting the scope of conclusions that can be drawn. Conversely, by normalizing the load to a certain percentage of each individual’s 1RM (e.g., 80% 1RM), then all would have exerted similar relative effort, thereby facilitating the uncoupling of abilities from relative effort (e.g., [76]).

#### Absolute and Relative Effort in Different Actions

The distinction between absolute and relative effort also allows for the examination of diverse effort expenditure pathways in various actions. Depending on the action, one can exert low absolute but high relative effort, or high absolute and relative effort, among other combinations. To illustrate, consider a person performing a maximal voluntary isometric contraction (MVC) with a hand gripper. The small muscle mass involved in the MVC requires less energy and thus low absolute effort. However, it requires maximal relative effort since the person exerts their maximal effort in performing the action. Should the same protocol be applied with larger muscle groups, like the quadriceps, it would require higher absolute effort, while the relative effort would remain maximal (e.g., [77]). This example highlights how absolute and relative effort can lead to different research questions and conclusions.

Notably, researchers focusing on physical effort assess maximal performance and derive relative effort more frequently than those focusing on cognitive effort. This tendency could be partially attributed to the relative ease of measuring maximal performance in various physical tasks. For example, MVCs are considered a valid and reliable measure of maximal force production and can be assessed in minutes [78, 79]. In contrast, it can be more challenging to quantify maximum cognitive performance, and it is certainly more difficult to prescribe relative cognitive effort (e.g., what would 80% of maximum cognitive effort be?). Regardless of the reasons, this situation implies that physical effort studies currently provide a more nuanced way to study relative effort compared to cognitive effort studies.

### Time

When measuring effort, it is essential to be explicit about the time units of interest. To do so, we defined energy with respect to the total or rate of energy used, enabling focus on either instantaneous or cumulative effort. By instantaneous effort, we refer to the amount of energy utilized per unit of time, aligning with outcomes such as force, velocity, and power. Conversely, cumulative effort refers to the sum of resources exerted throughout the activity, aligning with outcomes such as impulse, displacement, and work. Note that in contrast to mechanical variables like force, velocity, power, and their integrals, energy utilization (for effort) cannot be negative.

To illustrate the importance of being explicit about the time unit of interest, consider a resistance exercise set composed of numerous repetitions. One can measure force in a particular repetition (instantaneous) or the total force, or impulse, exerted throughout the set (cumulative). Similarly, during a complex math problem, one can measure effort during individual steps, such as when performing straightforward algebraic manipulations (instantaneous) or after solving the problem as a whole (cumulative). Considerable differences may be observed between the two measurements in both examples.

## Perception of Effort (PE)

When individuals exert effort, they often experience PE. The study of PE has led to distinctive advancements and challenges within the physical and cognitive effort fields. In the following discussion, we provide a brief overview of prevalent viewpoints on PE within each field and propose a definition of PE that overcomes some identified issues. We then discuss PE as a variable and cover important qualitative and quantitative measurement aspects. Definitions of key PE-related terms, which will now be covered, can be found in Table 2.

**Table 2:** Definitions of key perception of effort terms

Term	Definition
Perception of effort	The instantaneous experience of utilizing energy to perform an action
Rating of perceived effort (RPE)	Numeric assessment of perception of effort by way of single-item scales
Instantaneous RPE	RPE concerning an instant in time
Cumulative RPE	RPE concerning an extended period, typically a full training session
Imposed anchor	An anchor representing a specific action that is selected by an external source to the rater (e.g., researcher or coach). The rater then provides their RPE relative to that action
Self-selected anchor	An anchor representing a self-selected action, commonly the greatest effort the rater has ever experienced or one they can imagine. The rater then provides their RPE relative to that action

### PE in the Physical Domain

Within the physical domain, numerous PE definitions have been proposed [1, 9, 80–82]. We provide two highly cited definitions as examples. Borg [81] defined PE as “The feeling of how heavy and strenuous a physical task is,” emphasizing that “PE depends mainly on the strain and fatigue in your muscles and on your feeling of breathlessness or aches in the chest”. Noble and Robertson [82] defined PE as “The subjective intensity of effort, strain, discomfort and/or fatigue that is felt during exercise.” Several articles critically examined common PE definitions and identified two principal issues: circularity and inadequate differentiation between PE and other perceptual experiences [9, 11, 13].

Circularity arises when definitions of PE incorporate terms like laborious, strenuous, and exertion, which may be seen as synonymous with effort, thereby causing confusion and overlap. Inadequate differentiation arises when PE definitions include terms such as heavy, fatigue, pain, and discomfort. Including such terms is problematic as it has been established that individuals can differentiate PE from these experiences [83–85]. Other proposed definitions bypass some of these difficulties [9, 13, 80], including the one we will introduce in Sect. 6.3.

### PE in the Cognitive Domain

In the cognitive domain, PE is often interpreted using cost–benefit models, suggesting that PE reflects the costs of an ongoing task compared to its benefits or the benefits of engaging in other activities (i.e., opportunity cost) [4, 5, 20, 43, 86]. According to these models, cognitive actions like attention and memorization are limited and can be only allocated toward one task at a time. Therefore, PE is assumed to motivate disengagement from an activity when the perceived costs outweigh the perceived benefits.

Analyzing the cost–benefit equation in relation to PE allows us to draw several conclusions. First, PE aligns with the cost part of the equation. Second, the benefit part relates to the evaluation of ongoing and alternative opportunities. While this evaluation process may require effort, it is mainly aligned with shifting one’s motivation to persist with an action and is thus not a defining component of PE. Third, when the evaluation process reveals an appealing alternative opportunity, resisting the temptation of switching to it can be considered effortful [87]. For example, completing math drills (or reading this paper) with a smartphone nearby introduces a dual demand: (1) utilize energy to perform the drills and (2) resist distractions. Focusing on the drills and resisting distractions arguably demands more effort than solely focusing on the drills [87].

A narrow definition of PE focusing on the cost of performing an action may better capture the experiential aspects of interest. However, the narrowness of our definition has potential limitations worthy of consideration (see Sect. 9).

### Developing a Definition of PE

In view of the above (Sects. 6.1 and 6.2), our proposed definition of PE aims to (1) bypass the circularity and inadequate differentiation issues and (2) maintain a narrow meaning consistent with our definition of effort. Accordingly, we define PE as *the instantaneous experience of utilizing energy to perform an action*.

Since the proposed PE definition is an extension of our definition of effort, only a few explanations are required. To clarify our use of the term “instantaneous,” we draw on Daniel Kahneman’s distinction between the “experiencing self” and the ‘remembering self’ [88–90]. The “experiencing self” relates to one’s real-time, ongoing experience. In contrast, the “remembering self” involves a retrospective process of recalling, integrating, and assessing past experiences before evaluating them. These two selves can result in substantial differences in evaluations of comparable situations [90, 91]. The term “instantaneous” better aligns with the “experiencing self,” emphasizing the immediacy of PE that can be reported in real time. Notably, some PE researchers collect cumulative PE evaluations of an entire session (i.e., session rating of perceived effort [RPE]) akin to the “remembering self.” We will address these methodological aspects in Sect. 7.

While measurements of PE and effort tend to be associated [92–94], several factors can modify these associations [77, 95–97]. Notwithstanding these relationships, it is important to clarify early on that our rationale for this PE definition is not based on the assumption that PE reflects effort. Instead, our justification revolves around the definition of PE being a natural extension of the definition of effort. This definition is also narrow enough to exclude other experiences distinct from effort. Finally, we contend that our definition of PE is consistent with everyday language, making it easier to relate to and understand. Common expressions like “putting in effort,” “pouring your heart into it,” and “giving it all you have got” are aligned with our resource-based definition of PE.

### PE as a Variable

#### PE as a Latent Variable

Like effort, we regard PE as an unobservable latent variable. While both effort and PE are latent variables, we argue that the latent nature of PE is more pronounced. This is because effort can be inferred using objective measures (e.g., heart rate) without requiring the performer to actively communicate it. In contrast, since PE is a subjective experience, it should not be inferred until the individual explicitly reports it. The measurement tools used to assess PE are limited in their ability to capture PE as a private experience. Factors like the delay between exertion and the individual’s articulation of their PE, along with language and memory constraints, can impair the completeness and accuracy of such reports.

#### Operationalization of the Definition of PE

Since we conceptualize PE as a latent variable, our definition of PE also requires operationalizing. This can be done using a range of qualitative and quantitative tools (see Sect. 7). Regardless of the approach, the individual must describe or rate their PE. Hence, when defining PE to the raters, it is important to clearly explain and contextualize it (e.g., the term action) before collecting their responses. Such clarifications help the raters better understand what they are assessing within the context of a study or routine evaluation procedures.

#### PE as a Dependent or Independent Variable

Like effort, PE is commonly employed as a dependent variable. Researchers examine the effects of physical [76] or cognitive [98] interventions on participants’ reported PE. However, PE can also be conceived as an independent variable, for example, guiding study participants to run at a speed corresponding to a specified RPE value (see Sect. 7.1). Such manipulations tend to be viewed as related to the elicited running speed rather than the RPE leading to it. Once again, we are not suggesting changing this practice but believe there is some value in conceptually considering PE as an independent variable.

## Measurement Considerations

In this section, we review research tools aiming to capture PE. We begin with quantitative methods (or numerical relations) and move on to qualitative methods. We cover methodological considerations, how to extract the most from these tools, and their suitability for specific study designs.

### Quantitative Methods

The most common way to quantitatively assess PE is with single-item scales. In exercise science, this is done using RPE scales that commonly use ranges such as 0–10, 6–20, or 0–100 [81, 82, 99, 100], whereas cognitive effort researchers tend to use traditional Likert scales [98, 101]. Here, we cover the importance of carefully selecting and then consistently using the same definition of PE and instructions. Like effort, we selected these measurement aspects due to their relevance to our perspective. For a more comprehensive review, see Halperin and Emanuel [11].

#### Definitions

An ambiguous and/or overly broad definition of PE can negatively affect the interpretability of the reported ratings [9, 11, 13]. For instance, it is challenging to interpret a reported RPE value if the rater was provided with a PE definition that includes the terms fatigue or pain. To avoid confusion and ensure that PE is distinguished from other perceptual experiences, we suggest providing participants with a precise and narrow definition of PE, such as the one proposed here (Sect. 6.3).

#### Instructions

##### Instantaneous and Cumulative RPE

Once participants are familiar with the definition of PE, it is critical to ensure that they understand what they are rating, including whether their rating concerns instantaneous or cumulative RPE (analogous to instantaneous and cumulative effort). Instantaneous RPE refers to a specific moment and is relatively straightforward to report. We use the term instantaneous to denote a relatively fast response, but not an actual instantaneous one. This is due to the lag between experiencing PE and the time it takes to articulate it. Conversely, cumulative RPE refers to a longer period and can be more challenging to report. Session RPE is a form of cumulative RPE that is widely used to assess the RPE of an entire training session [102–104]. While considered reliable and valid [102], it is not always evident how trainees determine their rating. For instance, do they include or exclude inactive periods, and do they assign different weights to the peak or most recent effort experienced?

Researchers can minimize ambiguity by explicitly instructing participants to rate instantaneous or cumulative RPE. To illustrate, in tasks with multiple repetitions, vague instructions could lead participants to report their RPE in relation to a single repetition or the entire set. Without explicit instructions, both interpretations are valid and can influence the ratings. This clarification is particularly important when investigating the associations between effort and PE, as vague instructions could bias the strength of these associations—e.g., reporting cumulative RPE for an instantaneous effort. One approach that can partially bridge the gap between instantaneous and cumulative RPE is temporal experience tracing, in which individuals visually map their experience intensity over time on an empty graph [105], or, alternatively, continuous ratings made in real time [106].

##### Anchors

Another critical instructional aspect is the scale’s upper anchor (e.g., the meaning of 100 on a 0–100 scale). Comparison-based theories postulate that individuals cannot generate ratings in isolation [107–109]. Instead, individuals compare the experience of interest to reference points. Individuals may thus provide different ratings for the same effort, depending on the anchor. Here we differentiate between two types of anchors: imposed and self-selected. Imposed anchors represent a specific action that is selected by an external source to the rater (e.g., researcher or coach). Self-selected anchors are selected by the rater and are commonly defined as the greatest effort the rater has ever experienced or one they can imagine.

To illustrate, Malleron et al. [77] had participants perform repeated MVCs and report their RPE after each repetition. On one day, the anchor was imposed on a specific exercise, and on the other day, participants self-selected the anchor. Despite similar heart rates and forces, participants reported higher RPEs in the imposed anchor condition. Similarly, Morina et al. [110] had participants report their well-being using an 11-point scale relative to different anchors (e.g., previous-self, future-self, different person, etc.). Participants reported higher well-being when anchored to their previous compared to their future selves. Employing specific anchors can assist in exploring the relationship between physical and cognitive PE. For example, individuals can rate physical and cognitive actions relative to either a physical or cognitive imposed anchor. Understanding how physical actions are rated relative to cognitive anchors, and vice versa, can provide insight into how individuals perceive, relate, and weigh physical and cognitive efforts.

#### Strengths and Weaknesses of Quantitative Methods

Single-item RPE scales have a wealth of benefits. They are straightforward, easy to administer, and are considered reliable and valid. RPE scales are particularly useful for time-restricted studies and for collecting multiple real-time data during complex and physically demanding tasks. However, this simplicity is at the cost of being unable to capture other aspects of the PE experience. Single-item scales are also commonly implemented with researchers imposing a definition of what PE is and how it should be rated without considering participants’ viewpoints and understanding of PE. This may lead to participants providing ratings they may not fully understand or relate to. A valuable and underutilized strategy to overcome these limitations is qualitative methods.

### Qualitative Methods

Qualitative research, including interviews and focus groups, is designed to provide a deep and detailed account of phenomena using non-numerical data [111]. Compared to single-item scales, there is a noticeable lack of research employing qualitative techniques in studying PE. This contrasts with the field of pain research, where qualitative techniques have led to substantive theoretical developments and practical insights [112–115]. For example, using qualitative techniques revealed that when individuals rate their pain using single-item scales, they incorporate various dimensions of pain, including its duration, location, and intensity levels [114]. People also have difficulties rating their pain over specific time frames, such as the preceding 24 h, and articulating the basis behind their ratings [115]. Qualitative methods may greatly enhance our understanding of PE and should be implemented more frequently.

#### Qualitative Methods and PE

Analogously to pain research, qualitative methods can be beneficial for PE research. Understanding how people interpret existing PE definitions and their unique perspectives on such constructs could help refine PE definitions, improve instructions, and stimulate further inquiry into the topic. Qualitative work may also further our understanding of cumulative PE, for which individuals may struggle to provide RPEs. As illustrated previously (Sect. 7.1.2.1), how individuals rate session RPE remains unclear. Generating answers to such questions may lead to methodological adjustments in collecting session RPE. Yet, qualitative methods can be time-intensive, require skilled administration, and are challenging to analyze and compare across individuals [116]. We thus recommend combining both quantitative and qualitative methods, even in the same study, to gain the benefits from both worlds [117].

## Linking Effort and PE

Studying effort and PE concurrently can lead to important theoretical developments and practical insights. Theoretically, it allows us to explore how different factors account for performance—is it effort, PE, a blend of both, or do these factors vary based on the task or individual? Practically, we can investigate if PE can be manipulated such that individuals maintain constant effort levels while experiencing lower PE, thereby extending their performance boundaries. Furthermore, in cases where PE sufficiently reflects effort, tracking PE via RPE offers an accessible method to monitor and adjust physical activity levels. The factors discussed and detailed in this article can assist in addressing such questions (see Tables 1 and 2).

To illustrate, a common study design in exercise sciences involves participants completing a physical task while the researchers systematically adjust the task’s difficulty. Participants are asked to report their RPEs during or after the task [76, 92–94, 100]. Studies have identified moderate to very strong associations between RPE and effort indicators [76, 92–94, 100]. We contend that a substantial fraction of the variation in these associations may result from limited attention to the factors we covered, including the selection of the most suitable measures of effort (instantaneous vs. cumulative; absolute vs. relative), the employed definition of PE, and the clarity of RPE reporting instructions (instantaneous RPE vs. cumulative RPE; imposed vs. self-selected anchors). By carefully considering these factors, researchers can enhance the accuracy of their findings, thereby deepening our understanding of the interplay between effort and PE.

## Strengths and Weaknesses of this Perspective

Our perspective has specific weaknesses that warrant consideration. First, some might consider our perspective of effort excessively broad. However, such an expansive perspective could improve interdisciplinary communication. Second, our definition of PE is intentionally narrow to help distinguish PE from other experiences. However, this may be at the risk of being overly narrow, meaning it may exclude relevant perceptual experiences of PE that should be included in the definition. Moreover, whether individuals can effectively communicate the PE we defined here is also unclear, but this does not preclude the definition we put forward from being of theoretical interest. Employing qualitative methodologies could illuminate these matters, offering opportunities for further refinement of the definition. Third, certain assumptions of our perspective are still debated. Specifically, the role of depletable resources in cognitive effort has been contested [4, 42–44]. These ongoing debates highlight the need for continued research to refine and expand our understanding of the topic.

Adopting our perspective may offer several benefits. First, the proposed definitions of effort and PE are straightforward, clear, and conceptually related. Second, researchers from various fields can use this framework as a shared language, enabling them to bridge gaps and collaborate more effectively. The comparison between cognitive and physical effort throughout this paper highlights potential areas for exploration for both research fields. Third, this perspective can assist researchers in conducting and interpreting studies on effort and PE in a more organized, precise, and consistent manner.

## Conclusion

Despite the extensive research and conceptualization of effort and PE, discordant definitions between and within fields hamper scientific progress. Here, we have proposed an integrated perspective that offers definitions of effort and PE that apply to both physical and cognitive domains. We reviewed their characteristics as variables and covered important measurement aspects. We hope this perspective provides researchers with a comprehensive way to approach their research endeavors by sharpening effort-related constructs, fostering interdisciplinary collaborations, and enhancing research methodologies.

## Appendix: Linking Our Resource-based Account to Force- and Mediation-based Accounts of Effort

Here, we briefly expand on how our resource-based account of effort relates to—and more specifically, underlies—force- and mediation-based accounts of effort. Note that these concepts are best understood after reading Sect. 3 in its entirety, including the concepts of effective and ineffective effort introduced in Sect. 3.3.

Recall that we categorized effort into effective, which aids in achieving a goal, and ineffective, which does not. However, not all ineffective efforts are the same. In this section, we introduce two subcategories of ineffective effort: (1) neutral ineffective efforts (or simply neutral efforts), which use energy but neither advance nor hinder goal achievement, and (2) adverse ineffective efforts (or simply adverse efforts), which use energy and impair goal achievement by way of impairing performance. Building on these definitions, we also introduce the concept of net effective effort, which is the difference between the magnitudes of effective effort and adverse effort.

Force-based accounts contend that effort is best conceptualized as the force exerted to overcome a resistance force to achieve a goal. Massin [118] primarily focuses on mechanical efforts (cf. so-called “mental forces”). There are two broad mechanisms by which organisms can generate forces: (1) passively, which does not require energy that the organism expends ATP, e.g., from ligaments or muscles’ parallel elastic elements, and (2) actively, which necessitates that the organism expends ATP, e.g., actin-myosin cross-bridging. Our account does not consider the former, passive forces, to be effort as they do not require that the organism expends energy. However, since energy utilization underpins active force production, our resource-based definition of effort gives rise to force-based accounts of effort arising from active forces.

Importantly, not all active forces are analogous. Consider a unilateral isometric knee extension task aiming to maximize a net knee extension moment. While performing this task, one will produce effective forces by activating their knee extensors to create a knee extension moment. The individual may also simultaneously exert both types of ineffective effort. Neutral efforts may come in the form of motor overflow, i.e., activation of the contralateral knee extensors, which would not affect ipsilateral net knee extension moments. Adverse efforts could come in the form of co-contraction of the ipsilateral knee flexors, subtracting from the net knee extension moment. The force-based account presented above results from the combination of these effective and ineffective efforts: The net effective effort gives rise to the net force that the individual uses to interact with the world—e.g., to maximize their net knee extension moment—to accomplish their goal.

Mediation-based accounts state that effort mediates performance outcomes. By reconceptualizing this idea into our framework, it is possible to see that mediation-based accounts must reflect the net effective effort. Again, consider the above example of the knee extension task. If the individual’s goal is to maximize their net knee extension moment, then we should consider what mediates their performance toward this goal: Effective efforts positively mediate performance; neutral efforts have no effect on performance; and adverse efforts negatively mediate performance. Energy underlies these muscle actions and, thus, goal-oriented performance. Since net effective effort mediates performance and mediation-based accounts state that effort is what mediates performance, mediation-based accounts map onto our conceptualization of net effective effort.

## Footnotes

1 In this article, we use the term “effort” but equate it with “exertion.” Although some have proposed that nuanced differences might exist between these terms [1], we [11] and others [9, 13] view them as synonymous. This viewpoint is supported by the similar definitions of these terms in various languages and dictionaries and their interchangeable usage within exercise science [9].

2 We decided to use the term “cognitive” rather than “mental” because “cognitive” is assumed to have a neurophysiological basis, whereas “mental” tends to be associated with the spiritual mind, distinct from the physical body [18].

3 Generally speaking, more direct measures, such as calorimetry, are more desirable as they are more closely aligned with our effort definition and require fewer assumptions.
